# Using Ecological Indicators and a Decision Support System for Integrated Ecological Assessment at Two National Park Units in the Mid-Atlantic Region, USA

**DOI:** 10.1007/s00267-014-0391-y

**Published:** 2014-11-05

**Authors:** Carolyn G. Mahan, John A. Young, Bruce J. Miller, Michael C. Saunders

**Affiliations:** 1 The Pennsylvania State University, 209 Hawthorn Building, Altoona, PA USA; 2 U. S. Geological Survey, Leetown Science Center, Kearneysville, WV USA; 3 11817 Cedar Mill Road, North East, PA USA; 4 The Pennsylvania State University, 514 ASI Building, University Park, PA USA

**Keywords:** Decision support system, Ecological assessment, Landscape condition, National park, Water quality, Biological indicators, Ecological thresholds, Natural resource management

## Abstract

We implemented an integrated ecological assessment using a GIS-based decision support system model for Upper Delaware Scenic and Recreational River (UPDE) and Delaware Water Gap National Recreation Area (DEWA)—national park units with the mid-Atlantic region of the United States. Our assessment examined a variety of aquatic and terrestrial indicators of ecosystem components that reflect the parks’ conservation purpose and reference condition. Our assessment compared these indicators to ecological thresholds to determine the condition of park watersheds. Selected indicators included chemical and physical measures of water quality, biologic indicators of water quality, and landscape condition measures. For the chemical and physical measures of water quality, we used a water quality index and each of its nine components to assess the condition of water quality in each watershed. For biologic measures of water quality, we used the Ephemeroptera, Plecoptera, Trichoptera aquatic macroinvertebrate index and, secondarily, the Hilsenhoff aquatic macroinvertebrate index. Finally, for the landscape condition measures of our model, we used percent forest and percent impervious surface. Based on our overall assessment, UPDE and DEWA watersheds had an ecological assessment score of 0.433 on a −1 to 1 fuzzy logic scale. This score indicates that, in general, the natural resource condition within watersheds at these parks is healthy or ecologically unimpaired; however, we had only partial data for many of our indicators. Our model is iterative and new data may be incorporated as they become available. These natural parks are located within a rapidly urbanizing landscape—we recommend that natural resource managers remain vigilant to surrounding land uses that may adversely affect natural resources within the parks.

## Introduction

An ecological assessment is a science-based process and tool for determining the condition of an ecosystem relative to an identified condition or state (Jensen and Bourgeron 2001; Suter 2001; Levin et al. 2006). Ecological assessments, often based on monitoring data, are used for making or re-evaluating land management and/or regulatory decisions and reporting those decisions to the public (Bourgeron et al. 2001). These assessments have been adopted by many natural resource managers at both governmental and non-governmental organizations (Jensen and Bourgeron 2001; Suter 2001). These types of assessments do not primarily address causation of condition but summarize the status and trends of selected indicators (Suter 2001). The status and trend of the indicators are often compared against a reference condition (i.e., the natural structure and function of an ecosystem in the absence of any human impacts) for reporting ecosystem health (Egan and Howell 2001; Stoddard et al. 2006).

Indicators are measures that describe the condition of an ecosystem and/or its components (e.g., pH, biological indices, species diversity). For each indicator, ecological thresholds (e.g., the point at which there is an abrupt change in ecosystem quality or function) may be used to evaluate deviation from reference condition (see Groffman et al. 2006 for a review). Ecological thresholds have been used to show how pollution, land use change, or hydrological variability affects aquatic and terrestrial ecosystems and their components (Toms and Lesperance 2003; Rosenberg et al. 2004; Groffman et al. 2006; Lacoul and Freedman 2006). However, there are limited applications of thresholds across geographic regions and ecosystem type (Groffman et al. 2006).

In the mid-Atlantic region of the United States (USA), threshold indices for birds (O’Connell et al. 2000), macroinvertebrates (Klemm et al. 2003), and fish (Van Snik Gray et al. 2005) have been developed to show when a particular watershed or ecosystem has been ecologically impaired. Furthermore, water quality thresholds for temperature, pH, and dissolved oxygen (among others) have been recommended for streams in many geographic regions and landscape uses (see Brabec et al. 2002 for summary).

Landscape or habitat thresholds also have been suggested for forested ecosystems in the mid-Atlantic. For example, some studies determined thresholds for percent area in impervious surface and forest cover to maintain ecological health in a watershed (see Andrén 1994; Brabec et al. 2002; Schueler et al. 2009). However, assigning single value thresholds to landscape metrics to denote ecological integrity is a difficult task as species respond differently to habitat fragmentation across scales (Fahrig 2001; Tierney et al. 2009). Kennedy et al. (2003) found threshold values for proportion of suitable habitat remaining in the landscape ranging from 5 to 80 % for birds, 6–15 % for mammals, and 20–60 % for invertebrates. Similar variability in reported thresholds is evident for minimum habitat patch area, forest edge influence, and riparian buffer widths (Kennedy et al. 2003; King et al. 2005). Remaining habitat area (Andrén 1994), habitat quality (Fahrig 2001), and habitat fragmentation (Fahrig 2002) interact in complex ways to influence species distribution and extinction risk (Hanski 2011; Pardini et al. 2010). As a result, in spite of the wealth of landscape pattern metric indicators generated and evaluated over the past 20 years (McGarigal et al. 2009; McGarigal and Marks 1995; O’Neill et al. 1988, 1999; Utz et al. 2009; Uuemaa et al. 2009; Vogt et al. 2007), there are limited applications of these thresholds across geographic regions and ecosystem type.

Similar to defining ecological thresholds, determining reference condition has been attempted in a variety of ways. For example, natural areas (including National Parks) have been used to set reference conditions due to the presumably low human impacts in these areas (Lisle et al. 2007; Stephens and Fule 2005; Van Snik Gray et al. 2005). Furthermore, if historic conditions are known, these are often set as the reference condition for a particular area (Muxika et al. 2007; Stoddard et al. 2006; White and Walker 1997). However, for many ecosystems, reference condition(s) are simply not known.

In 2006, the National Park Service (NPS) implemented the Natural Resource Condition Assessment (NRCA) program to determine the current ecological state for natural resources as compared to reference condition (Fancy et al. 2009). The goal of these ecological condition assessments is to use a subset of indicators and thresholds to evaluate ecological condition of selected park units to frame management issues and alternatives and to report these findings to the public and government stakeholders. One limiting aspect of this assessment program is only existing data may be used—no data are collected as part of the NRCA efforts. In addition, specific management issues or goals are not identified for these assessments a priori.

Under this directive, we conducted an NRCA for the Delaware Water Gap National Recreation Area (DEWA) and Upper Delaware Scenic and Recreational River (UPDE)—national park units located in the mid-Atlantic (USA). We approached our assessment in a step-wise fashion, loosely following the objectives outlined in Levin et al. (2006). These objectives included:

- Determining the scale of the ecological assessment and datasets to use
- Developing a general reference condition for our study area
- Selecting ecological indicators to represent our ecosystem and associated thresholds
- Using selected ecological indicators and thresholds both individually and in an integrated fashion to assess ecosystem condition.

Following these objectives, we developed and applied an indicators-based decision support system (DSS) model that incorporated geographic information systems (GIS), fuzzy logic, expert judgment, best available data, and ecological thresholds to assess the ecological components of watersheds at UPDE and DEWA in 2010. A DSS refers to the use of a decision maker’s own insight integrated with computer information processing capabilities for improving quality of decision making (Turban 1993; Varma et al. 2000). This approach also involves integrating data from a variety of sources that may differ in scale and form. However, a DSS is not an automated decision maker. Management decisions are left to natural resource expertise.

The General Management Plan for UPDE and DEWA specifies that these parks were established for the protection of the “free-flowing Delaware River [that] cuts through a narrow valley, and the adjacent lands [that] contain streams and waterfalls, geologic features, a variety of plants and wildlife, and cultural resources (NPS 1987).” With the parks’ purpose in mind, we integrated both aquatic (biotic and abiotic) and landscape components and assessed ecological condition quantitatively and graphically. Although DSS models have been developed for forested landscapes (Varma et al. 2000) and aquatic resources (Mysiak et al. 2005), we believe ours is one of only a few ecological assessments that integrate indicators and associated thresholds from different ecosystem components into a unifying DSS model at the watershed and sub watershed level (see Tran et al. 2002, Sheldon et al. 2012). Our approach incorporates selected ecological thresholds and components that were available to us. However, we are not advocating our particular model, indicators, or thresholds. Rather, we demonstrate that an ecological assessment of place can be facilitated, and long-term management enhanced, through the codification and delivery of extant knowledge of broad ecological systems to resource managers and other stakeholders.

### Study Area

**Fig. 1:**
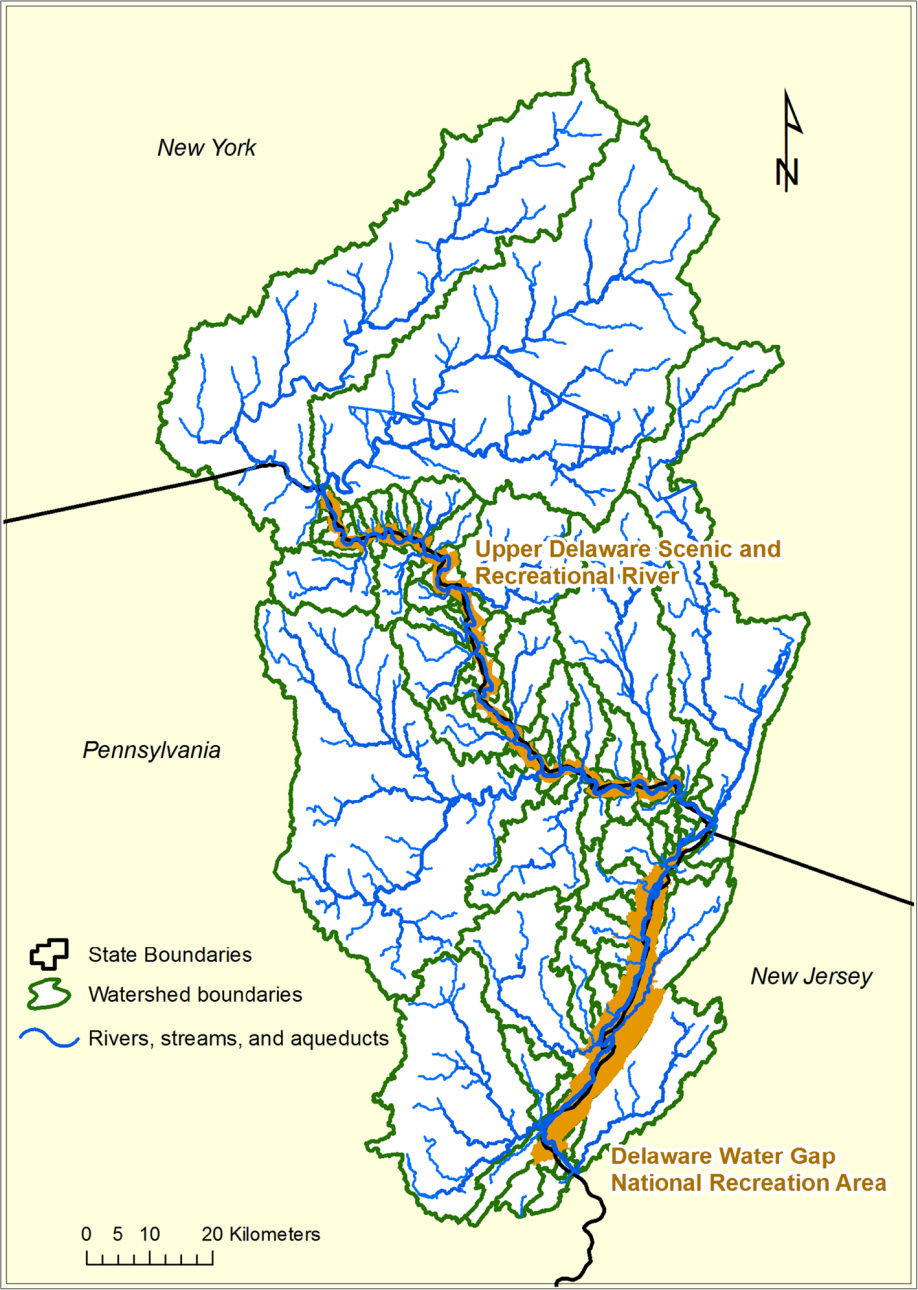
Location, watershed boundaries, and park boundaries of Delaware Water Gap National Recreation Area and Upper Delaware Scenic and Recreational River, 2009

We conducted our study at UPDE and DEWA, which are national park units located in northeastern Pennsylvania (PA), southern New York (NY), and northwestern New Jersey (NJ) within the glaciated low plateau physiographic province, USA (Fig. 1). The authorized boundary of UPDE encompasses 22,316 ha of riparian and riverine environments straddling the Delaware River in northeastern Pennsylvania and southern New York, but currently has only 12.5 ha in NPS ownership. The natural features of note within this park unit are related to the river itself and include outstanding game fish habitat, diverse native aquatic insect communities, and intact riparian plant communities. DEWA encompasses 27,762 ha of forested hills, ravines, and bottomlands bordering the Delaware River. Approximately, 21,885 ha of DEWA is forested; of this total, 18,575 ha is hardwood forest, 1,295 ha is coniferous forest, and 2,015 ha is mixed evergreen-deciduous forest (Young et al. 2002). Elevation within the parks ranges from 84 to 490 m (Snyder et al. 2002). At UPDE and DEWA, summer (June–August) air temperatures average 24–29 °C with low temperatures to 10 °C at night. Winter air temperatures (December to March) are frequently below freezing (0 °C), with numerous snow and ice storms in some years. Fall and spring temperatures are highly variable. Precipitation is evenly distributed throughout the year with annual amounts ranging between 86.4 and 132.0 cm (Knight et al. 2012). Due to the linear nature of these park units, threats to their natural resources arise primarily from outside the park and are associated with increased residential and commercial development.

## Materials and Methods

### Selecting Assessment Scale and Datasets

When conducting an ecological assessment, the scale of assessment must be consistent with the ability to recognize and explain drivers and threats to the ecosystem (Jensen and Bourgeron 2001; Levin et al. 2006; Serveiss 2002). We, therefore, used watersheds as the basis of conducting our ecological assessment at the two parks. These watersheds included the 100 major and minor tributaries present within, or flowing into, each park in addition to small direct surface runoff areas flowing into the main stem of the Delaware River. Watersheds were the logical scale at which to direct model development because they naturally incorporate aquatic and terrestrial indicators, are ecologically meaningful, and lend themselves well to comparisons with other natural resource agency programs (e.g., Environmental Protection Agency [EPA], US Forest Service, Delaware River Basin Commission, NY Department of Environmental Conservation). In addition, many datasets were already being collected at the watershed level within each park. By using the topographically defined watershed approach, our dataset contained a mix of catchment areas and stream orders (watershed areas ranged from 1.37 to 2,175.45 sq km). Accordingly, we focused on landscape metrics that are not sensitive to spatial extent.

Geospatial data used for determining watershed boundaries and all other georeferenced landscape data were from NPS datasets or national mapping programs using moderate resolution (~1:100,000 scale) digital elevation data, Landsat satellite imagery-based interpretations, and/or aerial photography-based interpretations (~1:12,000 to ~1:20,000 scale). The finer scale data sources are generally restricted to within park boundaries rather than inclusive of the entire watershed. Therefore, moderate resolution satellite-based land cover maps (e.g., the National Cover Dataset) were used as a primary data source for this assessment, and finer scale aerial photography-based land use and vegetation cover maps were incorporated where possible.

To conduct our assessment, we used a variety of ecological datasets that were available for park management. The project specifications limited us from collecting new data to conduct the assessment. To identify existing sources of scientific data and information useful for evaluating the current condition and trends of natural resources, all relevant reports and publications were identified by using NatureBIB (a National Park Service reports database), by cooperation with resource managers at park units, and by directly contacting researchers and organizations (e.g., Delaware River Basin Commission [DRBC]) who have conducted projects pertinent to natural resources in the parks. In addition, we relied on recently completed conceptual model reports for terrestrial ecosystems, major rivers, and tributaries that were available from the NPS Eastern Rivers and Mountains Network environmental monitoring program (e.g., Marshall and Piekielek 2005). Sources of data included, but were not limited to DRBC special waters program, water quality and quantity monitoring programs, published groundwater, natural resource, and recreational studies (e.g., Kauffman et al. 2011). Another important source of raw water quality data for many watersheds was USGS river gage data (http://waterdata.usgs.gov/pa/nwis/rt).

### Determining General Reference Condition and Indicators

As previously stated, there are a variety of ways to determine reference condition; however, we relied primarily on past scientific research and expert opinion in the same manner that we used for identifying sources of data. We supplemented these queries with two, ½-day workshops held at the parks so that consensus could be reached on reference condition and ecological thresholds. The workshops were attended by NPS and US Geological Survey (USGS) biologists, hydrologists, and geographers housed at the individual parks as well as biologists and ecologists who staff the NPS Eastern Rivers and Mountain Inventory and Monitoring Network. These workshops permitted resource managers to affirm the use of selected indicators and datasets.

In many ecological studies, a well-defined and documented reference condition is not available in the scientific literature (e.g., we do not know water quality, quantity, or some species assemblages [macroinvertebrates] of the historic [pre-Columbian] landscape of UPDE and DEWA). However, numerous landscape and biological changes (e.g., species introductions) indicate that UPDE’s and DEWA’s current condition does not reflect reference condition. In general, the reference and desired condition of the parks is a mixed deciduous and eastern hemlock (*Tsuga canadensis*) forest with cold freestone headwater streams (1st and 2nd order) originating in the park, and larger tributaries flowing through the parks (NPS 1987). In addition, a major feature of both parks is the Delaware River, which is the largest continuously flowing (undammed) river in the eastern United States (NPS 1987). Riitters et al. (2002) demonstrate that this geographic area has a large amount of intact, interior forest relative to other areas in the mid-Atlantic region. Because the reference condition of the park units is a forested landscape containing numerous cold water streams and an undammed river, we selected indicators to represent water quality (biologic and chemical/physical components) and landscape condition of this type of landscape within the mid-Atlantic USA (Table 1). These three types of indicators (1) chemical and physical indicators of water quality (biological oxygen demand [BOD], dissolved oxygen [DO], fecal coliform, nitrate, temperature change, total suspended solids, total phosphate, pH, and turbidity, (2) biologic indicators of water quality (macroinvertebrate indices), and (3) landscape condition (percent forest and percent impervious surface in a watershed) were combined within our model to provide an overall assessment of ecological condition of each watershed and, overall, each park.

**Table 1:** Components, selected indicators, source of information, and thresholds for watershed condition assessment of Delaware Water Gap National Recreation Area and Upper Delaware Scenic and Recreational River, 2009

Components	Selected indicator	Source	Thresholds
Chemical and physical water quality	Water Quality Index (WQI); includes biological oxygen demand [BOD], dissolved oxygen [DO], fecal coliforms, nitrate, temperature change, total suspended solids, total phosphate, and turbidity	Kaurish and Younos (2007)	90–100, excellent
			70–90, good
			50–70, medium
			25–50, bad
			0–25, very bad
Biologic water quality	Ephemeroptera, Plecoptera, and Trichoptera index (EPT)	Plafkin et al. (1989)	>27 indicates excellent water quality
			1–27 good water quality
			14–20 good–fair water quality
			7–13 fair water quality
			0–6 poor or ecologically impaired water quality
	Hilsenhoff Biologic Index (HBI)	Hilsenhoff (1987)	0.00–3.50 excellent
			3.51–4.50 very good
			4.51–5.50 good
			5.51–6.50 fair
			6.51–7.50 fairly poor
			7.51–8.50 poor
			8.50–10.00 very poor
Landscape condition	Percent forest in watershed	Andrén (1994), Kennedy et al. (2003)	Continuous range from <30 % (impaired) to >70 % (ideal)
	Percent impervious surface in watershed	Schueler and Holland (1994)	Continuous range from 0 % (ideal) to 10 % (impaired)

### Chemical and Physical Indicators of Water Quality and Thresholds

Several commissions and agencies have attempted to evaluate the chemical and physical measures of water quality in and around the parks. For example, the DRBC uses 14 measures to determine standards of existing water quality for the Delaware River mainstem; however, these measures are not associated with thresholds and are only used to describe current condition of the mainstem water quality. Furthermore, the USGS, in cooperation with the NJ Department of Environmental Protection (DEP), evaluated surface water quality in and around UPDE and DEWA by examining nine measures. These measures were compared to water quality standards for the state of NJ in conjunction with the EPA, which are consistent with the protection of aquatic life or drinking water standards. Such standards are mirrored by NY Department of Environmental Conservation (DEC) and PA Department of Environmental Protection (DEP). In fact, all states have set some water quality standards that are based upon values using threshold concentration (TEC) below which adverse effects on aquatic organisms (plants, fish, invertebrates) and aquatic sediment dwelling organisms are not expected to occur. These thresholds, however, are not necessarily reflective of the reference condition for streams in and around UPDE and DEWA. However, established water quality measures do provide standards with which to compare water quality within and around the parks and to examine trends in water quality.

Kaurish and Younos (2007) developed a standardized water quality index (WQI) for evaluating and reporting surface water quality that combines nine water quality measures (biological oxygen demand [BOD], dissolved oxygen [DO], fecal coliform bacteria, nitrate, temperature change, total suspended solids, total phosphate, pH, and turbidity) into a single interpretable value. To calculate the WQI, an indicator’s value (e.g., pH 7.0) is converted to a Q-value (0–100 point scale). The 100-point Q-value scale is non-linear, continuous, and based on conditions suitable for fish species found in the mid-Atlantic region of the United States (see Kaurish and Younos 2007 and Mahan et al. 2011 for individual Q-value scales). These Q-values are combined for all indicators to produce the WQI. The 100-point WQI can be divided into several score ranges corresponding to general descriptive terms for water quality (Table 1). In recent years, WQIs have been used to evaluate water quality in a variety of geographic areas (Qian et al. 2007; Bhatti and Latif 2011; Semiromi et al. 2011).

### Biologic Indicators of Water Quality and Thresholds

Biologic integrity in the context of waterways is defined as the ability of a water body to support and maintain a balanced, integrated, adaptive community of organisms having a species composition, diversity, and functional organization comparable to that of a natural habitat of the region (Daniels et al. 2002; Karr 1991; Southerland et al. 2007, Sponseller et al. 2001). In waterways, biological integrity has been measured using biotic indices related to macroinvertebrates, fish, and aquatic plant communities (Ladson et al. 1999; Sponseller et al. 2001; Lacoul and Freedman 2006). At UPDE and DEWA, we chose not to use fish indices (although they have been developed for PA and the mid-Atlantic–see Van Snik Gray et al. 2005) because it was difficult to determine what the natural community of fish in UPDE and DEWA should be due to the long-term introduction of non-native species (e.g., 60 % of the fish present in the parks are non-native) and the desire for park units to maintain these non-native populations for recreational use. Furthermore, we did not have adequate aquatic plant data or established thresholds to use this indicator in our assessment.

Rather than fish or aquatic plants, we chose to use the stream macroinvertebrate indices, Ephemeroptera, Plecoptera, and Trichoptera (EPT), and Hilsenhoff Biologic Index (HBI) to assess the biological integrity of waterways at the parks. EPT taxa richness is the number of taxa from the insect orders Ephemeroptera, Plecoptera, and Trichoptera. These orders are generally considered pollution sensitive, and EPT index values are usually depressed in polluted ecosystems (Wallace et al. 1996). The EPT index can range from 0 to the maximum number of EPT taxa encountered. Generally, an EPT index >27 indicates excellent water quality; while an index <6 indicates poor water quality. The HBI uses the relative organic pollution tolerance of all macroinvertebrate taxa and their relative abundance to assign a numerical value to aquatic communities. As opposed to the EPT index, the HBI value ranges from 0 to 10 with lower values indicative of a community dominated by highly sensitive organisms and high values indicative of dominance by pollution-tolerant organisms (Table 1; Hilsenhoff 1987; Plafkin et al. 1989). The choice of these indices was based upon research completed by the Academy of Natural Sciences in UPDE and DEWA (Patrick Center for Environmental Research 2001). This bioassessment study examined the relationship between stream macroinvertebrates and microhabitat characteristics as well as examining the correlation among indices used to assess biological integrity of waterways. Their findings indicated that the EPT index was the best index of habitat condition and ecological integrity within the park watersheds (Patrick Center for Environmental Research 2001). However, if the data to inform the EPT index were not available, the HBI was used as an alternative (or secondary) assessment.

### Indicators of Landscape Condition and Thresholds

We used the modeling software ATtILA: Analytical Tools Interface for Landscape Assessment to generate landscape condition metrics of UPDE and DEWA watersheds (Ebert and Wade 2004). ATtiLA generates numerous potential metrics for assessing the condition of terrestrial resources. However, previous studies have noted the high degree of redundancy in landscape configuration metrics and have used correlation analysis and factor analysis to determine metrics that provide unique information (Cifaldi et al. 2004; Kearns et al. 2005; King et al. 2005; Riitters et al. 1995). Additionally, several studies (Kearns et al. 2005; Saura and Martinez-Millan 2001) warn against using pattern metrics that are sensitive to spatial extent (i.e., those that vary in relation to size of the watershed under study), as these metrics are not good discriminators of landscape structure between catchments that vary in size.

Given these caveats (landscape metric redundancy and spatial sensitivity of metrics), we reviewed the literature and examined the science-based evidence for selecting landscape thresholds to denote ecological integrity (Tierney et al. 2009; Swift and Hannon 2010). Kennedy et al. (2003) distilled the range of reported landscape thresholds and cautiously recommended using percent forest thresholds for area sensitive species that we adopted in this study. In particular, the percent of intact forest in a watershed should be >70 % for ideal condition and, if the percent forest in a watershed falls below 30 %, then most species associated with our terrestrial reference condition would not be supported (e.g., King et al. 2005).

There is more agreement on ecological thresholds of urban/impervious land cover in mid-Atlantic watersheds, and several studies have found that negative effects to fish and macroinvertebrate communities are evident with urban/impervious land cover of 5–10 %, with serious impacts to community structure above 10 % (Schueler et al. 2009). In a review of impervious land cover effects on streams, Schueler and Holland (1994) propose a three-level classification system for stream condition based on percent urban/impervious area; sensitive streams (0–10 % impervious), impacted streams (11–25 % impervious), and impaired streams (26–100 % impervious).

Percent intact forest and percent impervious surface have accepted thresholds that are not directly related to landscape area making them ideal landscape indicators for our study. The thresholds selected for these indicators are <30 % is unacceptable and >70 % is ideal for percent intact forest, and the threshold values for impervious surface ranges from 0 % (ideal) to 10 % (unacceptable)—thresholds for both indicators are evaluated on a continuous scale (Table 1).

### Development of Decision Support System (DSS) Models

We developed and applied a decision support system (DSS) model that integrated our selected indicators and thresholds to provide a comprehensive quantitative and graphic (geospatial) watershed-based assessment of the ecological condition of selected natural resources in the parks. Although the field of fuzzy logic began as a way to model language ambiguities, its ability to quantify ambiguity (e.g., sparse datasets, non-linear and continuous assessment thresholds) in a numerical sense has been supported in industrial and ecological applications (e.g., Chang and Chen 2001; Bojorquez-Tapia et al. 2002). DSS models are useful for ecological assessments because data from different domains, formats, and sources can be integrated to assist in management decisions and understanding (Rausher and Potter 2001; Recknagel 2011). To develop our DSS models for natural resource condition assessments at UPDE and DEWA, we used NetWeaver™, an object-oriented software application developed at Penn State University (Paterson et al. 2008; Porter et al. 2006; Saunders et al. 2005). As a stand-alone tool, NetWeaver has been used to evaluate lake water chemistry (Saunders et al. 2005, Sullivan et al. 2005), and to evaluate watershed conditions (Reynolds et al. 2000). As a component of larger, integrated decision support systems with GIS capabilities, it has been used to address environmental issues such as carbon sequestration (Wang et al. 2010), wetlands management (Janssen et al. 2005), and wildland fire danger (Hessburg et al. 2008).

Our DSS incorporated fuzzy arguments that compared current condition of selected indicators against associated ecological thresholds for each indicator. Fuzzy statistical modeling integrates expert judgment with statistics of vague data and imprecise information to model ecological condition at multiple scales (Li 2001). The fuzzy argument compares the data values against a fuzzy set membership function that returns a level of trueness based on the degree of membership in the fuzzy set. In NetWeaver™, fuzzy set membership is measured on a scale of −1 (no membership in the fuzzy set “true,” which is equivalent to 100 % “false”), to 0 (“undetermined or neutral” in the case of insufficient data) to 1 (complete membership in the fuzzy set “true” which is equivalent to 100 % “true”). When the data associated with an application are spatially referenced, those data and the associated NetWeaver knowledge base can be displayed using the mapping capabilities of GeoNetWeaver™.

**Fig. 2:**
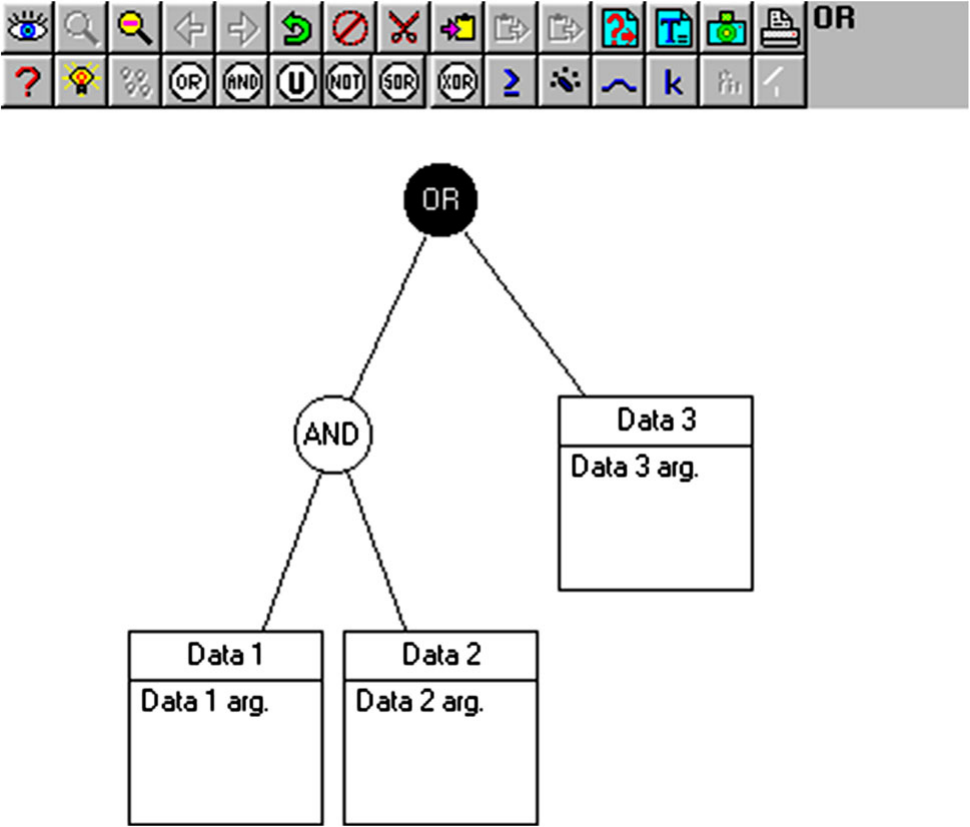
A dependency network as displayed in NetWeaver. In this dependency network, there are three data links represented by the *squares* at the *bottom* of the figure. Each of the data links evaluates the data value according to the extent to which it satisfies its arguments. The network can be read as a rule as follows: IF Data 1 satisfies the argument (arg.) “Data 1 arg.” AND Data 2 satisfies the argument “Data 2 arg.” OR Data 3 satisfies the argument “Data 3 arg.” THEN the assertion is true. The degree to which the assertion is true is a function of the degree(s) to which the individual data satisfy their arguments and the types and arrangements of the logical nodes used within the network

NetWeaver models consist of dependency networks. A dependency network is a graphical representation of a rule or syllogism (Fig. 2). Data that are entered into a NetWeaver model are first evaluated by data links. A data link can be a simple data link, which will compare the data value to an argument in order to assign a trueness level. Alternatively, some data may require mathematical manipulation and these data are evaluated by a calculated data link. Calculated data links also may have arguments to interpret the trueness level of the output of the calculation contained within it. Data links are at the bottom of dependency networks and are connected to the dependency network by logical nodes. In our model, these logical nodes are AND or OR (Fig. 2).

Arguments within data links can be decision thresholds that return a discrete value of true or false. Alternatively, a fuzzy argument can be used in which the data are compared to a fuzzy set (e.g., continuous values) membership function that issues trueness levels based on the degree of membership in the fuzzy set as defined by the argument.. For example, as shown in Table 1, several well-understood indicators are included in this model, including the EPT Index for biologic water quality. The fuzzy argument for interpreting the field value of EPT is a straight line or ramp from an EPT value of 6 to an EPT value of 27. Any EPT value at or below 6 will be assigned a fuzzy set membership value of −1, and any value at or above 27 will be assigned a value of 1. Intermediate values reflect intermediate levels of biological water quality on the continuum between −1 and 1. The use of fuzzy arguments to address the interpretation of environmental indicators greatly reduces the modeling effort and enhances the interpretation of indicator levels. For example, the various break points characterizing the intermediate levels used as thresholds in the original EPT Index are quantized to assist in their interpretation. There is a range of values in the EPT index that indicate “fair water quality” (i.e., 7–13) and a range of values indicating “good–fair water quality,” etc. However, interpreting and reporting an EPT Index as “fair water quality” fails to inform as to where on the “fair” scale the actual values fall. Is it a low fair value of 7 or a high fair value of 13? With a continuous fuzzy argument (a linear one in this case), all the intermediate interpretations are captured by referring to the EPT Index value’s membership in the fuzzy set “Excellent Water Quality.”

A dependency network calculates its membership in the fuzzy set true by evaluating the trueness level calculated by the data links and then passing that value to the logical node to which they are connected. The membership of a logical node is calculated differently for each type of node. At the top of a dependency network is an OR or an AND logical node. It is at this logical node that the overall fuzzy set membership of a dependency network is calculated. An OR node always takes on the trueness value of the most true antecedent. In contrast, an AND node calculates its trueness value using the formula:

$${\text{AND value}} = {\text{minimum score}} + \left( {{\text{weighted average}}{-}{\text{minimum score}}} \right)*\left( {{\text{minimum score distance to false}}/{\text{distance from false to true}}} \right)$$

In traditional fuzzy logic, an AND node assumes the fuzzy set membership value of the least true antecedent to which it is attached. In this modified approach, our NetWeaver model was run with partial or incomplete data. This ability to run the model with missing data permits the software to provide an interim evaluation based upon the data at hand, and permits the software to report to the user the rank order of missing data starting with the most important or influential of those missing data. This calculation of influence is based upon the topology of the network, and the values of the data already populating the model. This feature optimizes data collection and ensures that appropriate emphasis is placed on collecting the most important of the data that are missing.

## Results

### Model Output

**Fig. 3:**
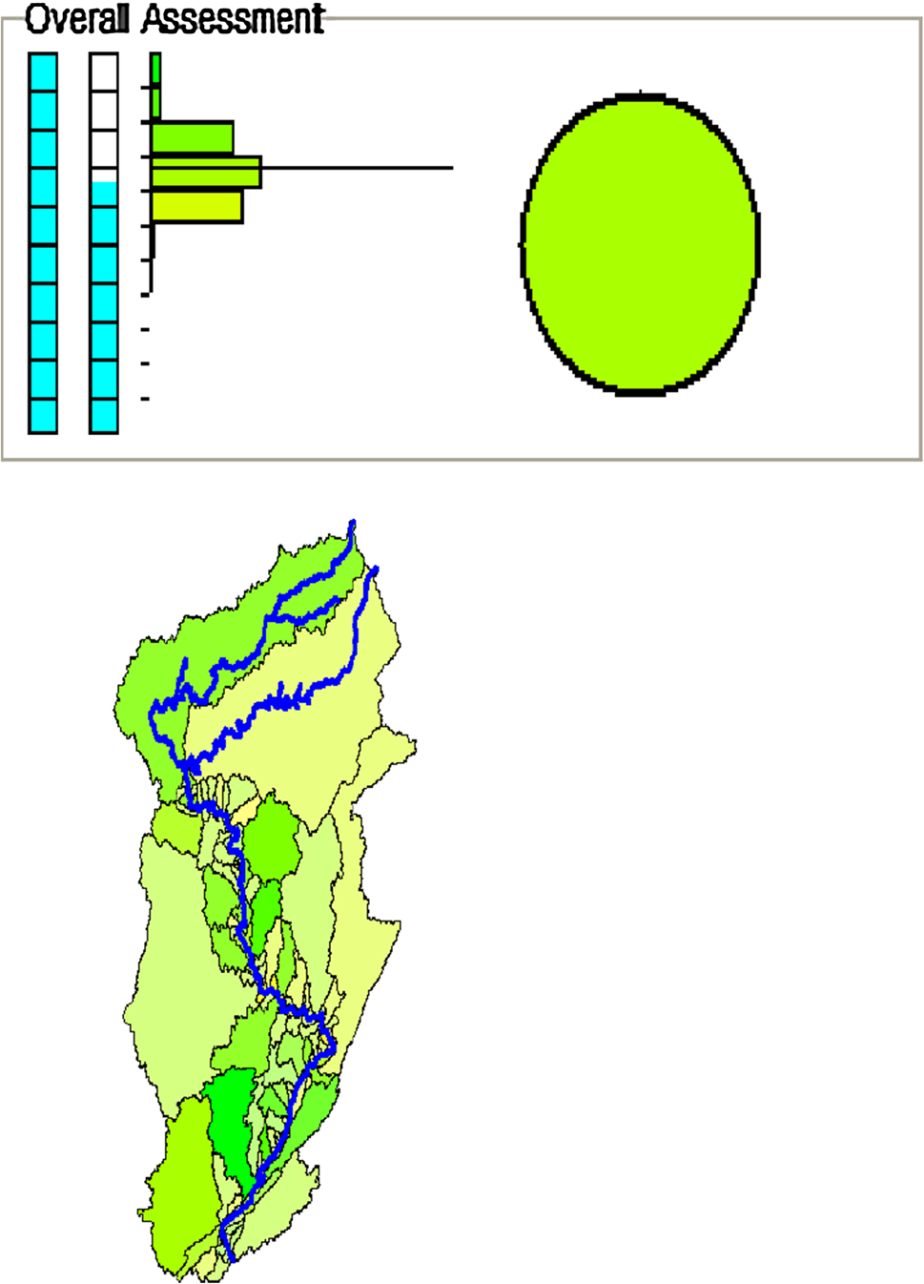
Sample ecological assessment model dashboard display demonstrating features that permit the reader a rapid understanding of assessment outcomes. The *left vertical bar* represents how much geographic area was used (sum of watershed areas with some data/sum of all watershed area). The *right vertical bar* represents the area-weighted average of data needs that were met (e.g., using only watershed *polygons* with some data). The *colors* used in the *histogram*, generated on a 1 to −1 fuzzy logic scale, represent poor quality (*red*), fair quality (*yellow*), and good quality (*green*). The brightness of the oval can be read to determine data sufficiency (e.g., a *dim color* indicates low data availability)

A feature of the graphical output of our model is a summary “dashboard” for the overall assessment and each indicator (Fig. 3). The dashboard displays a variety of features that permits the reader (e.g., public, government stakeholders) to gain a rapid understanding of the assessment outcomes. The dashboard displays two vertical bars. The left bar represents how much geographic area was used (sum of watershed areas with some data/sum of all watershed area). The right bar represents the area-weighted average of data needs that were met (e.g., using only watershed polygons with some data) so that a full bar would mean the model is completely populated with data and an empty bar would mean we had no data at all. The colors used in the histogram represent ecological condition on a 1 to −1 fuzzy logic scale: poor quality (red), fair quality (yellow), and good quality (green). The horizontal bars in the histogram indicate the relative amount of geographic area for each category of quality (e.g., the distribution of quality over the geographic area of the map). The colored oval represents the area-weighted average quality for the assessment or indicator. In addition, the brightness of the oval can be read to determine data sufficiency (e.g., a dim color indicates low data availability). Finally, the dashboard displays a qualitative watershed map with corresponding colors to graphically and rapidly indicate quality of individual watersheds for which we have data (Fig. 3).

### Data Availability

All watersheds had some data to complete the assessment; however, most had at least some incomplete data (Table 2). For example, completeness of data sources ranged from 100 % for the Toms Creek watershed to 48 % for the Heller Creek watershed in DEWA. This absence of complete datasets is due, in part, to uneven sampling across watersheds within the parks. In particular, macroinvertebrates were only sampled at a limited number of sites. For water chemistry, samples were not taken in all watersheds, nor did all watersheds have USGS gage data. In contrast to the gaps in aquatic measures, land cover data were available for all watersheds.

**Table 2:** Decision support system dependency networks and dashboard (described in text) output for overall watershed condition assessment, individual indicators, and associated thresholds

Statistical assessment results for ecological indicators and associated thresholds also are provided on a −1 to 1 continuous fuzzy logic scale for Delaware Water Gap National Recreation Area (DEWA) and Upper Delaware Scenic and Recreational River (UPDE), 2009

For both parks, 55 % of the data were available for calculating at least a partial WQI score (Table 2). However, the particular data available for calculating a WQI score differed among individual watersheds. For example, the Brodhead Creek watershed in DEWA had a previously calculated WQI score (82.8 out of 100; a good water quality rating)—therefore, our assessment model indicates that 100 % of data were available to calculate the score for that watershed. In contrast, for the Mongaup River watershed in DEWA, a partial score was calculated from existing data (USGS gage data) of which only 53 % are present. The resulting partial WQI was 63—an intermediate score for water quality. Therefore, due to these data gaps, we recommend that resource managers examine individual values for model indicators and not rely completely on overall scores.

For the aquatic biologic (macroinvertebrate) portion of the model, approximately 38 % of the watersheds in the parks had some data (Table 2). Complete macroinvertebrate datasets for calculating EPT or HBI were available for 23 watersheds. All of these datasets were found in published and unpublished technical reports and papers (Mahan et al. 2011).

Because landscape indicators were calculated from satellite imagery, all watersheds had complete datasets (Table 2). However, the data reflect the condition at the time that the images were originally acquired (2001). Therefore, there may be some differences between these data and current landscape condition of the parks.

### Watershed Assessment Outcomes

When all model indicators were combined as equally weighed parts of the DSS model, the overall score for the parks’ watersheds was 0.43 (based on the −1 to 1 fuzzy logic scale) (Table 2). Most indicators had multiple overall sources of data; however the data sources were not uniformly available over the area of study. The DSS model selected a data source for each metric, watershed by watershed, based on availability of data and a prioritization of data sources for each metric so that at each watershed the best data for that particular metric was used. In some cases, there were no data available at a watershed for a metric. In these cases, missing data were given a neutral score (zero) for purposes of propagating values up through the sub models. The lack of desired data was also tracked and reflected in graphical representation of watershed condition. Thus, data insufficiencies are monitored and noted but do not unduly handicap analysis.

For watersheds for which we had all or partial data, WQI scores, in general, indicate an overall high water quality at both parks (Table 2). High water quality was particularly evident for watersheds, where complete WQI scores were available. For example, the average score for watersheds with complete WQI scores was 82.4 (good but not excellent water quality). However, if the WQI scores (partial and complete) were averaged across both parks, the average WQI score is 63.1 (medium water quality) in part due to incomplete datasets (Table 2). Our model permits park managers to add additional data as it becomes available to complete the WQI for individual watersheds. Once more data are collected and added to the model, we expect the average WQI for all watersheds, individually and combined, to increase.

Based on available data, the overall assessment of biologic indicators was generally good (ecologically unimpaired); however, Brodhead Creek watershed in DEWA had an ecologically impaired EPT score of 6.7. Several pollution sources within this watershed (e.g., industrial pollution, human-induced development) may be influencing the low score. When the macroinvertebrate index scores (EPT or Hilsenhoff) are converted to the fuzzy logic scale (−1 [false—severely ecologically impaired] to 1 [true—pristine]), the biologic score average across both parks is 0.55 for those watersheds with some macroinvertebrate data—reflecting good condition (Table 2).

Based on percent forest and percent impervious surface in a watershed, the overall assessment of the landscape component of the model was uniformly good (Table 2). With the exception of the Port Jervis direct drainage watershed at UPDE, a small, heavily urbanized watershed (10.4 % impervious surface; 64.5 % forested) and the Jacoby Creek watershed in DEWA (2.28 % impervious surface; 49.8 % forested), both parks had a landscape component that indicates a good forest condition. When the landscape measures (percent forested, percent impervious surface) are converted to the fuzzy logic scale of the DSS model, the parks had an overall landscape model score of 0.92 (on the fuzzy logic −1 to 1 scale) which indicates excellent landscape condition (Table 2).

## Discussion

### Advantages and Drawbacks of the Model

Our data compilation and analysis resulted in the ability to assess natural resource condition over two national park units by summarizing chemical, biologic, and landscape indicators within watershed units in a DSS context, even where data were incomplete. By scoring indicators using a fuzzy logic scale, we were able to avoid hard decision thresholds, which can lead to abrupt transitions in assessment scores. Suter (2001) critiqued the tracking of indicators and associated indices to determine the condition of a site; rather he recommends reporting “real units of environmental properties” (e.g., response variables [pH, DO, species richness]). Our model permits resource managers to do both. Although the “top layer” of reporting is an overall condition score (or index), users may find and report data associated with particular environmental properties. For example, each component score for the WQI may be reported for watersheds for which we have data. Our model also contains the formula for calculating the EPT index so users can report the raw species richness data for each category of macroinvertebrate. Finally, the percent forest and percent impervious surface data are available for each watershed within the study area and may be reported as such if a resource manager is interested in a particular portion of the park(s).

Our model has some similarities to Tran et al. (2002) in that they developed a method to rank watersheds using fuzzy indicators and landscape variables—although they structured their analysis into a decision hierarchy using the Analytical Hierarchy Process (AHP). Although Tran et al. (2002) applied a Principal Components Analysis to select a hierarchy of ecological indicators, they did not formalize their fuzzy ranking methodology into a DSS. In contrast, we developed a method to integrate landscape indicators with field measured chemical and biologic data, while enabling scoring of watersheds with incomplete data. The scoring of watersheds with incomplete data provides a conservative estimate of ecosystem condition as missing data are treated as neutral by the model. Unlike Sheldon et al. (2012) who examined a large number of datasets to determine what scales and what measures can be reliably used to assess watershed condition, we used available (and often limited) data to determine what watersheds exhibit degradation. Our findings support other work in the Appalachians that indicate forest coverage can predict watershed health (King et al. 2005; Sheldon et al. 2012).

Our DSS allows national park units to incorporate available chemical and biological monitoring data into an integrative assessment of watershed condition. Accordingly, the logic rules used in our DSS are designed to take advantage of data that currently exist in park databases. Our model readily illustrates, where data gaps are present and helps resource managers prioritize inventory and research efforts at the parks. For example, data to better determine and monitor water quality (e.g., temperature change, flashiness, non-macroinvertebrate biotic indicators) were missing from most watersheds within the parks. In 2007, the NPS developed an ecological monitoring plan and data collected as part of this effort may help fill-in these gaps (Marshall and Piekielek 2007). Data that will be collected as part of this recent monitoring effort include hydrological parameters (velocity, discharge, and flood characteristics) at selected sites within each park (Marshall and Piekielek 2007).

The DSS approach permits model users to change the dependency networks, change the prioritization of data, and add or remove indicators and measures—thus, it is flexible and dynamic. The natural resource assessment compares certain measures with ecological thresholds and reference condition and can be updated as new thresholds are developed or as reference condition becomes better understood. In addition, the model can be re-run with new data or additional data so that natural resource managers can examine trends in natural resource condition over time. This ability to examine trends will help park managers set and address performance and reporting goals that must be developed under the Government Performance Results Act (GPRA 1992, 103 PL 62). Because the model is tied to datasets, the findings are defendable and the graphic interface facilitates reporting to the public.

Our menu-based, computerized model is multi-dimensional and non-linear which makes it difficult to translate into a linear written report. Such reports are the preferred medium (at this time) for result presentations by governmental agencies and scientific journals. We acknowledge that such a translation presents a difficult hurdle for adequately communicating the range of model outputs.

### Application of the Model

Ecological assessments facilitate understanding of a landscape’s past, present, and future condition in the face of natural and/or anthropogenic threats (Jensen and Bourgeron 2001). Our assessment provides a baseline against which the effects of many threats (e.g., urbanization, industrialization) and actions (e.g., park management decision, water quality regulations) can be measured. For example, in 2010, proposed regulations to protect water resources of the Delaware River Basin during the development and operation of Marcellus shale natural gas projects were proposed. Adoption of these regulations (or non-adoption) may affect water quality and forest cover around UPDE and DEWA (Kargbo et al. 2010). Our assessment provides a “pre-Marcellus shale natural gas extraction” condition of the Delaware River watershed within these national park units. Furthermore, NJ, NY, and PA state governments are working with the DRBC to develop Total Maximum Daily Loads (TMDLs) in order to improve or maintain water quality around UPDE and DEWA. Once TMDLs are implemented, indicators of water quality (chemical/physical and biologic) can be re-assessed to document any changes. Finally, Jantz and Morlock (2011) found that increasing population pressure and associated residential development have the potential to change ecosystem functions within park boundaries. In particular, human population density, impervious surface area, rural housing density, and agricultural land coverage outside the parks have all increased in the past 10 years (Riva-Murray et al. 2010). Goetz and Fiske (2008) found that landscape connectivity has declined around UPDE and DEWA and these trends may continue with current land use trends. Again, our assessment provides data for the park to document any negative effects on natural resources that may occur due to these increased pressures outside park boundaries.

## References

[CR1] Andrén H (1994). Effects of habitat fragmentation on birds and mammals in landscapes with different proportions of suitable habitat: a review. Oikos.

[CR2] Bhatti MT, Latif M (2011). Assessment of water quality of a river using an indexing approach during the low-flow season. Irrig Drain.

[CR3] Bojorquez-Tapia LA, Juarez L, Cruz-Bello G (2002). Integrating fuzzy logic, optimization, and GIS for ecological impact assessments. Environ Manage.

[CR4] Bourgeron PS, Humphries HC, Jensen ME, Jensen ME, Bourgeron PS (2001). Ecosystem characterization and ecological assessments. A guidebook for integrated ecological assessments.

[CR5] Brabec E, Richards PL, Schulte S (2002). Impervious surfaces and water quality: a review of current literature and its implications for watershed planning. J Plan Lit.

[CR6] Chang FJ, Chen YC (2001). A counterpropagation fuzzy-neural network modeling approach to real time streamflow prediction. J Hydrol.

[CR7] Cifaldi RL, Allan JD, Brown DG, Duh JD (2004). Spatial patterns in land cover of exurbanizing watersheds in southeastern Michigan. Landsc Urban Plan.

[CR8] Daniels RA, Bilger MD, Halliwell DB, Riva-Murray K, Vana-Miller DL (2002). An index of biological integrity for northern mid-Atlantic slope drainages. Trans Am Fish Soc.

[CR9] Ebert DW, Wade TG (2004) Analytical tools interface for landscape assessments (ATtILA): User Manual. EPA/600/R-04/083. U.S. Environmental Protection Agency, Office of Research and Development, National Exposure Research Laboratory, Environmental Sciences Division, Landscape Ecology Branch, Las Vegas, NV

[CR10] Egan D, Howell EA (2001). The historical ecology handbook: a restorationist’s guide to reference ecosystems.

[CR11] Fahrig L (2001). How much habitat is enough?. Biol Conserv.

[CR12] Fahrig L (2002). Effect of habitat fragmentation on the extinction threshold: a synthesis. Ecol Appl.

[CR13] Fancy SG, Gross JE, Carter SL (2009). Monitoring the condition of natural resources in US national parks. Environ Monit Assess.

[CR14] Goetz S, Fiske G (2008). Linking the diversity and abundance of stream biota to landscapes in the mid-Atlantic U.S.A. Remote Sens Environ.

[CR15] Groffman PM, Baron JS, Blett T, Gold AJ, Goodman I, Gunderson LH, Levinson BM, Paerl HW, Palmer MA, Peterson GD, Poff LN, Rejeski DW, Reynolds JF, Turner MG, Weathers KC, Wiens J (2006). Ecological thresholds: the key to successful environmental management or an important concept with no practical application?. Ecosyst.

[CR16] Hanski I (2011). Habitat loss, the dynamics of biodiversity, and a perspective on conservation. AMBIO.

[CR17] Hessburg P, Reynolds K, Keane R, James K, Salter R (2008). Evaluating wildland fire danger and prioritizing vegetation and fuels treatments. For Ecol Manage.

[CR18] Hilsenhoff WL (1987). An improved biotic index of organic stream pollution. Gt Lakes Entomol.

[CR20] Janssen R, Goosena H, Verhoevenb ML, Verhoevenb JTA, Omtzigta AQA, Maltby E (2005). Decision support for integrated wetland management. Environ Model Softw.

[CR21] Jantz C, Morlock L (2011) Modeling urban land use change in the upper Delaware River Basin. Final report to National Park Service, U.S. Department of the Interior, Washington DC

[CR22] Jensen ME, Bourgeron PS, Jensen ME, Bourgeron PS (2001). Introduction. A guidebook for integrated ecological assessments.

[CR23] Kargbo DM, Campbell DJ, Wilhelm RG (2010). Natural gas plays in the Marcellus shale: challenges and potential opportunities. Environ Sci Technol.

[CR24] Karr JR (1991). Biological integrity: a long-neglected aspect of water resource management. Ecol Appl.

[CR25] Kauffman GJ, Belden AC, Homsey AR, Sanchez JR (2011). Water quality trends in the Delaware River Basin (USA) from 1980 to 2005. Environ Monit Assess.

[CR26] Kaurish FW, Younos T (2007). Developing a standardized water quality index for evaluating surface water quality. J Am Water Resour Assoc.

[CR27] Kearns FR, Carter JL, Kelly NM, Resh VH (2005). A method for use of landscape metrics in freshwater research and management. Landsc Ecol.

[CR28] Kennedy C, Balch J, Wilkinson J (2003). Conservation thresholds for land use planners.

[CR29] King RS, Baker ME, Whigham DF, Weller DE, Jordan TE, Kazyak PF, Hurd MK (2005). Spatial considerations for linking watershed land cover to ecological indicators in streams. Ecol Appl.

[CR30] Klemm DJ, Blocksom KA, Davis WS, Fulk FA, Griffith MB, Herlihy AT, Hughes RM, Kaufmann PR, Peck DV, Stoddard JL, Thoeny WT (2003). Development and evaluation of a Macroinvertebrate Biotic Integrity Index (MBII) for regionally assessing Mid-Atlantic Highlands Streams. Environ Manage.

[CR31] Knight P, Imhoff K, Bahrmann C, Miller S (2012) Weather of Delaware Water Gap National Recreation Area and Upper Delaware Scenic and Recreational River: Eastern Rivers and Mountains Network summary report for 2011. Natural Resource Data Series NPS/ERMN/NRDS—2012/385. National Park Service, Fort Collins, Colorado

[CR32] Lacoul P, Freedman B (2006). Environmental influences on aquatic plants in freshwater ecosystems. Environ Reviews.

[CR33] Ladson AR, White LJ, Doolan JA, Finlayson BL, Hart BT, Lake PS, Tilleard JW (1999). Development and testing of an index of stream condition for waterway management in Australia. Freshw Biol.

[CR34] Levin PS, Fogarty MJ, Matlock GC (2006). White paper on integrated ecosystem assessments.

[CR35] Li BL, Jensen ME, Bourgeron PS (2001). Fuzzy statistical and modeling approach to ecological assessments. A guidebook for integrated ecological assessments.

[CR36] Lisle TE, Cummins K, Madej MA (2007) An examination of references for ecosystems in a watershed context: results of a scientific pulse in Redwood National and State Parks, California. In Furniss M, Clifton C, Ronnenberg K (Eds.) Advancing the Fundamental Sciences: Proceedings of the Forest Service National Earth Sciences Conference, San Diego, CA, 18–22 October 2004, PNWGTR-689, Portland, OR: U.S. Department of Agriculture, Forest Service, Pacific Northwest Research Station

[CR37] Mahan, CG, Miller BJ, Saunders MC, Young JA (2011). Assessment of natural resources and watershed condition for Delaware Water Gap National Recreation Area and Upper Delaware Scenic and Recreational River. U.S. Department of the Interior, National Park Service Natural Resource Report NPS/NER/NRR-2011/429

[CR38] Marshall MR, Piekielek NB (2005) ERMN vital signs monitoring program: phase II report. USDI. National Park Service

[CR39] Marshall MR, Piekielek NB (2007) Eastern Rivers and Mountains Network Ecological Monitoring Plan. Natural Resource Report NPS/ERMN/NRR—2007/017. National Park Service. Fort Collins, CO

[CR40] McGarigal K, Marks BJ (1995) FRAGSTATS: spatial pattern analysis program for quantifying landscape structure. USDA Forest Service General Technical Report PNW-GTR-351, Pacific Northwest Research Station, Corvalis, OR

[CR41] McGarigal K, Cushman SA, Tagil S (2009). Surface metrics: an alternative to patch metrics for the quantification of landscape structure. Landsc Ecol.

[CR42] Muxika I, Bald J, Borja A (2007). Using historical data, expert judgement, and multivariate analysis in assessing reference conditions and benthic ecological status, according to the European Water Framework Directive. Mar Pollut Bull.

[CR43] Mysiak J, Giupponi C, Rosato P (2005). Towards the development of a decision support system for water resource management. Environ Model Softw.

[CR44] National Park Service (NPS) (1987) General Management Plan Delaware Water Gap National Recreation Area. U.S. Department of Interior, Washington, DC

[CR45] O’Neill RV, Christensen SW, Dale VH, DeAngelis DL, Gardner RH, Graham RL, Jackson B, Krummel JR, Milne BT, Sugihara G, Turner MG, Zygmunt B (1988). Indices of landscape pattern. Landsc Ecol.

[CR46] O’Connell TJ, Brooks RP, Jackson LE (2000). Bird guilds as indicators of ecological condition in the central Appalachians. Ecol Appl.

[CR47] O’Neill RV, Jones KB, Riitters KH, Wickham JD (1999). Landscape pattern metrics and regional assessment. Ecosyst Health.

[CR48] Pardini R, de Arruda Bueno A, Gardner TA, Metzger JP, Prado PI (2010). Beyond the fragmentation threshold hypothesis: regime shifts in biodiversity across fragmented landscapes. PLoS One.

[CR49] Paterson B, Beytell B, Brown C, Demas F, Dunne TT, Lindeque P, Schinzel B, Stuart-Hill G, Tagg J, Underhill LG, Weaver C (2008). A fuzzy decision support tool for wildlife translocations into communal conservancies in Namibia. Environ Model Softw.

[CR50] Patrick Center for Environmental Research (2001). Bioassessment study for the Delaware Water Gap National Recreation Area and the Upper Delaware Scenic and Recreational River 1997–Year 3.

[CR51] Plafkin JL, Barbour MT, Gross SK, Hughes RM, Porter KD (1989) Rapid bioassessment protocols for use in streams and rivers: Benthic macroinvertebrates and fish. U.S. EPA, Office of Water Regulations and Standards. Washington, D.C., EPA/440-4-89-001

[CR52] Porter A, Hayden N, Sadek A (2006). Fuzzy geographic information systems for phytoremediation plant selection. J Environ Eng.

[CR53] Qian Y, Migliaccio KW, Yongshan W, Yuncong L (2007). Surface water quality evaluation using multivariate methods and a new water quality index in the Indian River Lagoon, Florida. Water Resour Res.

[CR54] Rausher HM, Potter WD, Jensen ME, Bourgeron PS (2001). Decision support for ecosystem management and ecological assessments. A guidebook for integrated ecological assessments.

[CR55] Recknagel F (2011). Ecological informatics: a discipline in the making. Ecol Inf.

[CR56] Reynolds K, Jensen M, Andreason J, Goodman I (2000). Knowledge-based assessment of watershed conditrions. Comput Electron Agric.

[CR58] Riitters KH, Hunsaker CT, Jackson BL, Jones KB, O’Neill RV, Timmins SP, Wickham JD, Yankee DH (1995). A factor analysis of landscape pattern and structure metrics. Landsc Ecol.

[CR59] Riitters KH, Coulston JW, Jones KB, O’Neill RV, Smith ER, Smith JH, Wade TG, Wickham JD (2002). Fragmentation of continental United States forests. Ecosystems.

[CR60] Riva-Murray K, Riemann R, Murdoch P, Fischer JM, Brightbill R (2010). Landscape characteristics affecting streams in urbanizing regions of the Delaware River Basin (New Jersey, New York, and Pennsylvania, U.S.). Landsc Ecol.

[CR61] Rosenberg R, Blomqvist M, Nilsson HC, Cederwall H, Dimming A (2004). Marine quality assessment by use of benthic species-abundance distributions: a proposed new protocol within the European Union Water Framework Directive. Marine Pollut Bull.

[CR62] Saunders MC, Miller BJ, Nash BL, Sullivan TJ, Tonnessen KA (2005). A knowledge-based approach for classifying lake water chemistry. Knowl-Based Syst.

[CR63] Saura S, Martinez-Millan J (2001). Sensitivity of landscape pattern metrics to map spatial extent. Photogramm Eng Remote Sens.

[CR64] Schueler TR, Holland HK (1994). The importance of imperviousness. Watershed Prot Tech.

[CR65] Schueler TR, Cappiella K, Fraley-McNeal L (2009). Is impervious cover still important? Review of recent research. J Hydrol Eng.

[CR66] Semiromi FB, Hassani AH, Torabian A, Karbassi AR, Hosseinzadeh Lotfi F (2011) Water quality index development using fuzzy logic: a case study of the Karoon River of Iran. Afr J Biotechnol 10:10125–10133

[CR67] Serveiss VB (2002). Applying ecological risk principles to watershed assessment and management. Environ Manag.

[CR68] Sheldon F, Peterson E, Boone ED, Sippel S, Bunn S, Harch BD (2012). Identifying the spatial scale of land use that most strongly influences overall river ecosystem health score. Ecol Appl.

[CR69] Snyder CD, Lemarie DP, Smith DR, Young JA (2002). Influence of eastern hemlock (*Tsuga canadensis*) forests on aquatic invertebrate assemblages in headwater streams. Can J Fish Aquat Sci.

[CR70] Southerland MT, Boward DM, Kazyak PF, Klauda RJ, Kline MJ, Morgan RP, Rogers GM, Stranko SA (2007). Improving biological indicators to better assess the condition of streams. Ecol Indic.

[CR71] Sponseller RA, Benfield EF, Valett HM (2001). Relationships between land use, spatial scale and stream macroinvertebrate communities. Freshw Biol.

[CR72] Stephens SL, Fule PZ (2005). Western pine forests with continuing frequent fire regimes: possible reference sites for management. J For.

[CR73] Stoddard JL, Hawkins CP, Johnson RK, Larsen DP, Norris RH (2006). Setting expectations for the ecological condition of streams: the concept of reference condition. Ecol Appl.

[CR74] Sullivan TJ, Saunders MC, Tonnessen KA, Nash BL, Miller BJ (2005). Application of a regionalized knowledge-based model for classifying the impacts of nitrogen, sulfur, and organic acids on lakewater chemistry. Knowl-Based Syst.

[CR75] Suter GW (2001). Applicability of indicator monitoring to ecological risk assessment. Ecol Indic.

[CR76] Swift TL, Hannon SJ (2010). Critical thresholds associated with habitat loss: a review of the concepts, evidence, and applications. Biol Rev.

[CR77] Tierney GL, Faber-Langendoen D, Gibbs JP, Mitchell BR, Shriver WG (2009). Monitoring and evaluating the ecological integrity of forest ecosystems. Front Ecol Environ.

[CR78] Toms JD, Lesperance ML (2003). Piecewise regression: a tool for identifying ecological thresholds. Ecology.

[CR79] Tran LT, Knight CG, O’Neill RV, Riitters KH, Smith ER, Wickham J (2002). Fuzzy decision analysis for integrated environmental vulnerability assessment of the mid-Atlantic region. Environ Manag.

[CR80] Turban E (1993). Decision support and expert systems: management support systems.

[CR81] Utz RM, Boward DH, Hilderbrand RH (2009). Identifying regional differences in threshold responses of aquatic invertebrates to land cover gradients. Ecol Indic.

[CR82] Uuemaa E, Antrop M, Mander Ü, Marja R, Roosaare J (2009). Landscape metrics and indices: an overview of their use in landscape research. Living Rev Landsc Res.

[CR83] Van Snik Gray E, Bennett RM, Ross RM (2005). Bioassessment of Fish Communities of the Upper Delaware River. Northeast Nat.

[CR84] Varma VK, Ferguson I, Wild I (2000). Decision support system for the sustainable forest management. For Ecol Manag.

[CR85] Vogt P, Estreguil C, Kozak J, Riitters KH, Wade TG, Wickham JD (2007). Mapping spatial patterns with morphological image processing. Landsc Ecol.

[CR86] Wallace JB, Grubaugh JW, Whiles MR (1996). Biotic indices and stream ecosystem processes: results from an experimental study. Ecol Appl.

[CR87] Wang J, Chen J, Ju W, Li M (2010). IA-SDSS: a GIS-based land use decision support system with consideration of carbon sequestration. Environ Model Softw.

[CR88] White PS, Walker JL (1997). Approximating nature’s variation: selecting and using reference information in restoration ecology. Restor Ecol.

[CR89] Young JA, Lemarie DP, Smith DR, Snyder CD (2002). A terrain-based paired-site sampling design to assess biodiversity losses from eastern hemlock decline. Environ Monit Assess.

